# Structured exercise interventions for women at high risk of or diagnosed with hypertensive disorders of pregnancy: a scoping review

**DOI:** 10.3389/fcvm.2026.1876680

**Published:** 2026-07-21

**Authors:** Ruizhe Jiang, Li Shu, Fan Yang, Simin Huang, Xiangwu Li, Xinxin Ye, Qixi Liu

**Affiliations:** 1 Greatteam Smart Sport Technology Innovation Center (Beijing) Co. Ltd., Beijing, China; 2 Department of Sports Science, Zhejiang University, Hangzhou, Zhejiang, China; 3 College of Physical Education, Anhui Normal University, Wuhu, Anhui, China; 4 School of Medicine, Shaoxing University, Shaoxing, Zhejiang, China; 5 School of Nursing, Fujian University of Traditional Chinese Medicine, Fuzhou, Fujian, China; 6 Department of Nursing, Mindong Hospital of Fujian Medical University, Fuan, China

**Keywords:** exercise intervention, hypertensive disorders of pregnancy, physical activity, pregnancy, scoping review

## Abstract

**Background:**

Hypertensive disorders of pregnancy (HDP) are one of the leading causes of maternal and fetal morbidity and mortality worldwide. Although prenatal physical activity is broadly recommended for uncomplicated pregnancies, evidence regarding exercise prescription, safety monitoring, and clinically relevant outcomes among women at risk of or diagnosed with HDP remains insufficiently mapped.

**Objective:**

To map the existing literature on structured exercise interventions for the prevention and management of HDP and to identify gaps for future research.

**Methods:**

This scoping review was conducted in accordance with PRISMA-ScR. Four electronic databases (PubMed, Embase, Web of Science, and the Cochrane Library) and two trial registries (the World Health Organization International Clinical Trials Registry Platform and ClinicalTrials.gov) were searched from inception to December 19, 2025. Eligible studies evaluated structured exercise interventions in pregnant women diagnosed with HDP or at high risk of developing HDP. Two reviewers independently screened studies and extracted data using a predefined form. Extracted information included study design, participant characteristics, intervention characteristics, outcomes, and findings.

**Results:**

Thirteen articles published between 2008 and 2025 were included. The included articles primarily involved two clinical contexts: women at high risk of HDP and women with established HDP. The interventions included aerobic exercise, resistance training, combined aerobic and resistance exercise, stretching, and yoga. Overall, most studies reported that structured exercise interventions may improve blood pressure control, reduce the risk of certain HDP-related events, and improve pregnancy and neonatal outcomes, endothelial function, and maternal psychological well-being. However, the current evidence remains limited by substantial heterogeneity in the prescription parameters of exercise interventions. In particular, the definition, classification, and monitoring of exercise intensity were insufficiently operationalized across studies. In addition, safety monitoring, exercise termination criteria, adherence, and intervention fidelity were inadequately reported, and some studies used statistical methods that did not fully account for pre–post or repeated-measures designs.

**Conclusions:**

Available evidence on structured exercise interventions for HDP is limited, heterogeneous, and insufficient to inform population-specific exercise prescriptions. Future trials should adopt more rigorous designs, clearly define and monitor exercise intensity, improve statistical approaches, and include safety, adherence, and clinically meaningful maternal and neonatal outcomes.

**Systematic Review Registration:**

https://doi.org/10.17605/OSF.IO/DPVH3, identifier 10.17605/OSF.IO/DPVH3.

## 1 Introduction

Hypertensive disorders of pregnancy (HDP) affect over 10% of pregnant women and are one of the leading causes of maternal and fetal morbidity and mortality globally (1–4). Preeclampsia is characterized by new-onset hypertension after 20 weeks of gestation, commonly accompanied by proteinuria and/or maternal organ dysfunction or placental involvement (5, 6). In severe cases, the condition may be complicated by renal, cardiac, pulmonary, hepatic, and neurological dysfunction; hematologic abnormalities; fetal growth restriction; stillbirth; and even maternal death (6, 7). Established risk factors for HDP include advanced maternal age; maternal comorbidities such as chronic kidney disease, chronic hypertension, and obesity; nulliparity; multiple gestation; and a history of HDP, as well as a family history of preeclampsia (8, 9). Women with a history of hypertensive disorders of pregnancy have a significantly higher risk of developing chronic diseases and multimorbidity later in life (10–12).

The prevention and management of HDP remain challenging. At the individual level, management approaches for HDP primarily encompass pharmacological therapy, nutritional supplementation, and lifestyle modifications (12, 13). Nevertheless, the optimal approaches for preventing and managing HDP, minimizing associated complications, and improving pregnancy outcomes have not yet been fully established and remain under active investigation (5, 14). Exercise, a cost-effective lifestyle strategy, is attracting increasing attention because of its relevance to blood pressure regulation, vascular function, and broader cardiometabolic health (15). Substantial evidence supports the effectiveness of exercise in reducing blood pressure among non-pregnant adults (16–18). Prenatal exercise was also associated with a lower incidence of gestational hypertension, gestational diabetes, and macrosomia (19–21). However, specific guidance regarding exercise for the prevention and management of HDP remains limited, although exercise has been widely incorporated into routine prenatal care across multiple guidelines (22–25).

Existing evidence suggests that exercise may have beneficial effects on HDP-related outcomes, such as improved endothelial function, reduced blood pressure, and lower incidence of gestational hypertension or preeclampsia (26–28). However, there remains a lack of systematic synthesis regarding target populations and the characteristics of interventions. This gap is relevant in the context of HDP, where women with different risk profiles, disease stages, and severity may have distinct clinical goals and intervention needs (5, 14). A broader synthesis is therefore needed to characterize how structured exercise interventions have been studied across different HDP-related clinical contexts and to identify gaps in the current evidence base.

Therefore, this scoping review aimed to map the existing literature on structured exercise interventions for the prevention and management of HDP, with particular attention to intervention characteristics, participant clinical profiles, disease severity, and outcome assessments, and to identify gaps for future research.

## 2 Methods

This scoping review was conducted and reported in accordance with the Preferred Reporting Items for Systematic Reviews and Meta-Analyses extension for Scoping Reviews (PRISMA-ScR) checklist (Supplementary Material S1) (29). For details of the pre-registered study protocol, see Open Science Framework: https://osf.io/dpvh3. A comprehensive electronic literature search was conducted. Relevant studies published from inception to December 19, 2025 were searched across four electronic databases: PubMed, Embase, Web of Science, and the Cochrane Library. In addition, two trial registries, the World Health Organization International Clinical Trials Registry Platform (WHO ICTRP) and ClinicalTrials.gov, were searched as supplementary grey literature sources. The retrieval strategies were based primarily on MeSH subject words and text words using the Boolean search terms “OR” and “AND” (Supplementary Material S2).

### 2.1 Eligibility criteria

The inclusion criteria were as follows: (1) studies used an interventional study design; (2) studies evaluated structured exercise interventions among pregnant women; structured exercise was defined as planned, structured, repetitive, and purposeful physical activity undertaken to improve or maintain one or more components of physical fitness; (3) the study population included pregnant women diagnosed with HDP or women at high risk of developing HDP; (4) the studies were published in English in peer-reviewed journals. Studies were excluded if they: (1) examined only acute physiological responses to a single bout of exercise or one-session intervention testing; (2) evaluated postpartum-only interventions; (3) included general pregnant populations without HDP risk factors, or HDP diagnoses; (4) had no full text available; or (5) were observational studies, reviews, protocols, conference abstracts, case reports, or other non-intervention studies.

Two researchers independently screened the titles and abstracts, retrieved the full texts of potentially relevant studies, and assessed their eligibility. Disagreements regarding the inclusion of studies were resolved through discussion. Data were extracted from the included records with a standardized, predefined form. The extracted data included: title, author, year of publication, study design, sample size, interventions, outcomes, and findings. Paper selection, screening, and data extraction were completed by March 4, 2026. Data were tabulated and summarized, and evidence gaps were identified. No critical appraisal or risk-of-bias assessment was conducted because the purpose of this scoping review was to map the extent, characteristics, and gaps in the available evidence rather than to determine intervention effectiveness.

## 3 Results

### 3.1 Study selection

**Figure 1:**
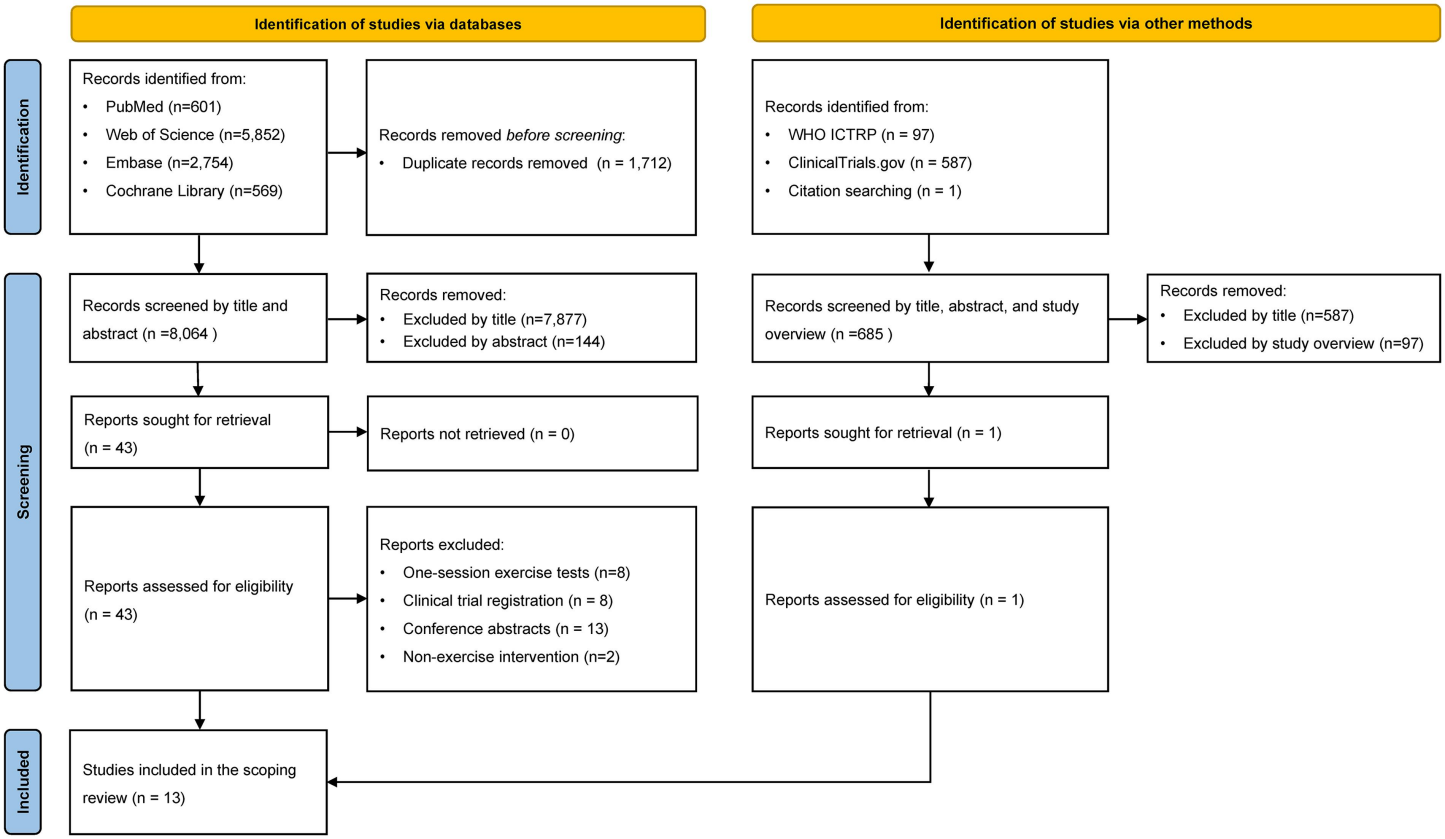
Flow diagram of study selection.

The systematic search revealed 10,461 potentially relevant records. After removing duplicates, 8,749 records were screened for eligibility. A total of 44 full-text reports were assessed against the eligibility criteria. Finally, 13 articles were included in this scoping review (Figure 1).

### 3.2 Characteristics of included articles

A total of 13 articles published between 2008 and 2025 were included. Most adopted randomized controlled trial (RCT) designs (*n* = 8) (28, 30–36), one was a quasi-experimental study (27), one was a non-randomized controlled study (37), and three were secondary analyses of previously conducted RCTs (26, 38, 39). Three articles were all derived from the same trial and reported different aspects of the outcomes (26, 30, 39). However, because the study by Yeo et al. (30) excluded participants who became ineligible, withdrew, or had missing data, the analyzed sample sizes differed across the three papers. The included studies were conducted in Indonesia (*n* = 1) (27), China (*n* = 3) (35–37), the United States (*n* = 4) (26, 30, 38, 39), India (*n* = 2) (33, 34), Egypt (*n* = 1) (32), Brazil (*n* = 1) (31), and Iran (*n* = 1) (28). (Table 1)

**Table 1:** General characteristics of the included studies.

Study	Country	Study design	Sample size
Prevention			
Yeo (2008) (30)	USA	RCT	Stretching, *n* = 38; Walking, *n* = 41
Yeo (2009) (39)	USA	Secondary analysis of RCT	Stretching, *n* = 60; Walking, *n* = 64
Yeo (2010) (26)	USA	Secondary analysis of RCT	Stretching, *n* = 60; Walking, *n* = 64
Khoram (2019) (28)	Iran	RCT	CG, *n* = 36; Walking, *n* = 36
Karthiga (2022) (34)	India	RCT	CG, *n* = 113; Yoga, *n* = 121
Zainiyah (2025) (27)	Indonesia	Quasi-experimental study	CG, *n* = 23; Yoga, *n* = 23
Claiborne (2025) (38)	USA	Secondary analysis of pooled RCTs	CG, *n* = 9; AE, *n* = 12; RE, *n* = 6; Combination, *n* = 10
Management			
Awad (2019) (32)	Egypt	RCT	CG (autogenic training), *n* = 20; Stretching, *n* = 20
Makhija (2021) (33)	India	RCT	CG, *n* = 30; Yoga, *n* = 30
Chen (2024) (35)	China	RCT	CG, *n* = 7; AE, *n* = 11; RE, *n* = 11; Combination, *n* = 11
Yu (2024) (36)	China	RCT	CG, *n* = 50; AE, *n* = 50
Wang (2025) (37)	China	Non-randomized controlled study	CG, *n* = 90; AE, *n* = 110
Mixed prevention and management			
Kasawara (2013) (31)	Brazil	RCT	CG, *n* = 53; Cycling, *n* = 56

CG, control group; AE, aerobic exercise; RE, resistance exercise; Combination, aerobic exercise + resistance exercise; RCT, randomized controlled trial.

### 3.3 Characteristics of included participants

 Table 2 presents the clinical and demographic characteristics of the participants. The included studies involved pregnant women with varying clinical profiles related to HDP. Nearly half of the included studies recruited women at high risk of developing HDP, including those with a previous history of preeclampsia, family history of hypertensive disorders, first pregnancy, obesity, advanced maternal age, or other established risk factors (26–28, 30, 34, 38, 39). One study included women with chronic hypertension and/or previous preeclampsia, representing a mixed population of women with established HDP and those at increased risk of HDP (31). The remaining studies included women who had already been diagnosed with HDP (32, 33, 35–37). One specifically recruited women with preeclampsia (32) and two focused on gestational hypertension (33, 37). Across the included studies, maternal demographic characteristics varied. The mean maternal age generally ranged from 25 to 35. In most trials, participants were recruited during mid to late pregnancy (approximately 14–32 weeks of gestation) (27, 28, 30–32, 34–36, 38), while one study did not report the gestational age at enrolment (37). In most studies, the mean parity generally ranged from approximately 0 to 2 previous births, while two studies included only primiparous women (32, 33).

**Table 2:** Characteristics of included participants.

Study	Population Clinical Characteristics	Maternal Age	Gestational Age	Parity
Prevention				
Yeo (2008) (30)	High-risk pregnant women (pregnant women were eligible if they: 1) had been diagnosed with preeclampsia during a previous pregnancy, 2) had a lower than average cardiovascular fitness level (i.e., peak oxygen consumption equal to or less than 50th percentile of age group women), and 3) had a sedentary lifestyle.)	CG: 20–34 (79%) EG: 20–34 (66.7%)	CG and EG: 18	CG: ≥1 EG: ≥1
Yeo (2009) (39)		CG: 20–34 (66.7%) EG: 20–34 (66.7%)	CG and EG: 18	CG: ≥1 EG: ≥1
Yeo (2010) (26)		CG: 31 ± 5 EG: 31 ± 5	CG and EG: 18	CG: ≥1 EG: ≥1
Khoram (2019) (28)	Pregnant women were susceptible to gestational hypertension and had a history of transient gestational hypertension, history of preeclampsia or eclampsia, chronic hypertension, autoimmune disease, history of transient hypertension or preeclampsia in a sister or mother, or obvious diabetes.	CG: 31.0 ± 5.3 EG: 31.9 ± 4.6	CG: 15.0 ± 1.4 EG: 14.0 ± 0.3	CG: 1.2 ± 1.1 EG: 1.6 ± 1.1
Karthiga (2022) (34)	The normotensive pregnant women before 16 weeks of pregnancy and having any of the established risk factors for GH, such as family history of preeclampsia or GH, preeclampsia or GH in previous pregnancy, extremes of reproductive age, first pregnancy, multiple pregnancy, etc.	CG: 23.6 ± 3.7 EG: 24.2 ± 3.8	CG: 16 EG: 16	NR
Zainiyah (2025) (27)	High risk of preeclampsia	CG: 27.9 ± 8.9 EG: 26.4 ± 8.7	CG: 14.8 ± 1.1; EG: 14.9 ± 1.0	CG: 2.4 ± 1.8 EG: 2.4 ± 2.0
Claiborne (2025) (38)	Risk of hypertensive disorders of pregnancy	CG: 29.6 ± 4.4 EG1: 28.4 ± 4.7 EG2: 30.7 ± 2.8 EG3: 29.7 ± 4.9	16	CG: 1 (0.2) EG1: 0 (0.2) EG2: 0 (0.1) EG3: 0 (0.2)
Management				
Awad (2019) (32)	Mild preeclampsia	CG: 29.0 ± 6.4 EG: 27.9 ± 7.3	CG and EG: exceeding 24	primipara
Makhija (2021) (33)	Mild gestational hypertension	CG: 25.9 ± 4.9 EG: 25.0 ± 3.0	CG: 242.8 ± 19.7 days (about 34) EG: 237.4 ± 12.9 days (about 33)	Primipara
Chen (2024) (35)	Hypertensive disorders of pregnancy	CG: 32.8 ± 3.6 EG1: 33.6 ± 5.9 EG2: 32.2 ± 3.4 EG3: 32.9 ± 5.2	24–32	CG: 0.2 ± 0.4 EG1: 0.3 ± 0.6 EG2: 0.4 ± 0.5 EG3: 0.5 ± 0.5
Yu (2024) (36)	Hypertensive disorder in pregnancy	CG: 28.4 ± 4.7 EG: 29.0 ± 4.1	CG: 31.4 ± 3.0; EG: 31.5 ± 3.1	CG: Primipara (62%) EG: Primipara (66%)
Wang (2025) (37)	Gestational hypertension	CG: ≤30 (48.9%) EG: ≤30 (46.4%)	NR	CG: ≤1 (80.0%) EG: ≤1 (78.2%)
Mixed prevention and management				
Kasawara (2013) (31)	Chronic hypertension and/or previous preeclampsia	CG: 20–39 (88.0%) EG: 20–39 (82.8%)	CG: 18.5 ± 3.4 EG: 17.3 ± 3.4	≥1 *n* (%) CG: 49 (84.5%) EG: 45 (77.6%)

CG, control group; EG, experimental group; NR: not reported.

### 3.4 Characteristics of structured exercise interventions

The characteristics of the interventions across the included articles are summarized in Table 3. A variety of exercise modalities were evaluated, including aerobic exercise, resistance training, combined aerobic and resistance exercise programs, stretching, and yoga.

**Table 3:** Characteristics of structured exercise interventions.

Study	Exercise type	Intervention duration	Frequency	Intensity	Time	Comparator	Termination criteria
Prevention							
Yeo (2008) (30)	Aerobic exercise (walking)	18 weeks’ gestation to delivery	5/week	Moderate	40 min	Stretching	Participants were instructed to stop or avoid exercise if warning signs occurred according to ACOG safety guidelines
Yeo (2009) (39)			5/week	Moderate	40 min	Stretching	
Yeo (2010) (26)			5/week	Moderate	40 min	Stretching	
Khoram (2019) (28)	Aerobic exercise (walking)	14–34 weeks’ gestation	4/week	Moderate	20–30 min	Routine prenatal care	The mothers were asked to refer to their physician and inform the researcher in cases they had signs of danger, such as shortness of breath, palpitations, difficulty, and imbalance while walking.
Karthiga (2022) (34)	Yoga	20 weeks	2/day	Low	30 min	Standard antenatal care	NR
Zainiyah (2025) (27)	Yoga	4 weeks	5/week	Low	30 min	Standard care	NR
Claiborne (2025) (38)	EG1: aerobic exercise EG2: resistance training EG3: combination	16 weeks’ gestation to delivery	3/week	moderate	50 min	Stretching/breathing control	NR
Management							
Awad (2019) (32)	Stretching	6 weeks	3/week	Low	NR	Relaxation procedures	NR
Makhija (2021) (33)	Yoga	4 weeks at least	3/week	Low	40 min	Routine physical activities and bed rest	NR
Chen (2024) (35)	EG1: aerobic exercise (walking) EG2: resistance training EG3: combination	≥4 weeks	3–5/week	Moderate	∼60 min	No exercise intervention	NR
Yu (2024) (36)	Aerobic exercise (walking)	NR	1/day	Moderate	30 min	Routine care	Stop if shortness of breath or palpitations occur
Wang (2025) (37)	Aerobic exercise (walking)	3 months	7/week	Moderate	30 min	Standard nursing care	NR
Mixed prevention and management							
Kasawara (2013) (31)	Aerobic exercise (stationary bicycle)	12–20 weeks’ gestation to delivery	1/week	Low-to-moderate intensity	30 min	Routine prenatal care	NR

CG, control group; EG, experimental/intervention group; HRmax, maximum heart rate; VO_2_, oxygen uptake; RPE, rating of perceived exertion; NR: not reported.

In prevention-oriented studies involving women at high risk of developing HDP but without a confirmed HDP diagnosis at baseline, aerobic exercise was the most frequently implemented modality (26, 28, 30, 38, 39). Other studies evaluated yoga or multi-arm exercise interventions, including aerobic, resistance, and combined exercise modalities (27, 34, 38). Aerobic exercise and multi-arm exercise interventions were generally prescribed at moderate intensity and were more consistent with conventional prenatal physical activity recommendations (26, 28, 30, 38, 39). Several studies used heart rate (26, 30, 38, 39), the Rating of Perceived Exertion (RPE) (26, 30, 38, 39), or the talk test (28, 38) to regulate or monitor exercise intensity. Yeo et al. defined the target intensity as 55%–69% of the age-predicted maximal heart rate (26, 30, 39). Another study prescribed a target maternal heart rate range corresponding to 60%–80% of maximal oxygen uptake (38). Regarding intervention frequency and session duration, the highest frequency was twice-daily yoga practice (34), whereas the remaining interventions generally ranged from three to five sessions per week (26–28, 30, 38, 39). Session duration mainly ranged from 20 to 50 min (26–28, 30, 34, 38, 39). Regarding safety procedures, four articles reported exercise termination criteria or warning symptoms requiring cessation, including palpitations, breathing difficulty, and imbalance (26, 28, 30, 39). Reporting of exercise-related adverse events was generally limited, with only one study explicitly reporting the absence of exercise-related adverse events (34).

In management-oriented studies involving women with established HDP, intervention modalities included stretching and yoga, as well as aerobic, resistance, or combined exercise (32, 33, 35–37). In terms of intensity, two studies implemented low-intensity stretching or yoga interventions (32, 33), whereas the remaining three studies used moderate-intensity aerobic, resistance, or combined exercise interventions (35–37). Two studies used heart rate to monitor or regulate exercise intensity, maintaining a target range of 100–120 bpm (35, 37), with one study combining heart rate monitoring with RPE, the talk test, and a fixed walking speed (e.g., 50 m/min) (35). Another study monitored physical signs such as mild sweating and fatigue (36). Regarding intervention frequency and session duration, the highest frequency was once daily (36, 37), whereas the lowest frequency was three sessions per week (32, 33). Session duration ranged from 30 min (36, 37) to approximately 60 min (35). Regarding safety procedures, only one study reported exercise termination criteria or warning symptoms requiring cessation, including palpitations and breathing difficulty (36). One study also measured blood pressure after exercise sessions (36).

One study included a mixed population of women with chronic hypertension and/or previous preeclampsia (31). This study implemented an aerobic exercise intervention, with intensity defined as maintaining heart rate 20% above resting values, with a ceiling of 140 beats per minute (31). The intervention was delivered once per week for 30 min per session. This study measured blood pressure both before and after exercise sessions and explicitly reported the absence of exercise-related adverse events (31).

Regarding intervention fidelity in all included studies, a range of strategies were adopted to enhance the consistency, standardization, and traceability of intervention delivery. Some studies used wearable devices, such as a pedometer or a triaxial accelerometer, to monitor participants’ daily physical activity, thereby providing objective information on intervention exposure (26, 30, 35, 39). Two studies required participants to complete diaries to track their activity practice (33, 34). To improve the standardization of home-based or unsupervised interventions, several studies provided exercise videos to help participants perform the prescribed movements as intended (26, 30, 35, 39). In addition, most interventions were delivered under partial or full professional supervision, such as by exercise instructors, yoga teachers, physical therapists, or research staff, to ensure intervention consistency (26, 27, 30, 32–34, 38, 39). Regarding intervention adherence, only a small number of studies reported adherence outcomes. Yeo et al. provided a clear description of changes in adherence across gestation in each intervention group (26, 30, 39). Another study reported the mean number of intervention sessions attended by participants in the intervention group (31).

### 3.5 Characteristics of outcomes and findings

Overall, the included studies covered maternal cardiovascular regulation, endothelial function, metabolic health, perinatal outcomes, and maternal psychological well-being. Among maternal outcomes, blood pressure was the most common indicator, with 10 articles reporting the effect of intervention on blood pressure (28, 30, 32–39). Other blood pressure-related variables included pulse pressure and resting heart rate (26, 39). A proportion of studies evaluated exercise interventions from a disease prevention perspective, focusing primarily on the incidence of hypertensive disorders during pregnancy, including gestational hypertension, preeclampsia, superimposed preeclampsia, and eclampsia (28, 30, 34).

Pregnancy and delivery outcomes were another major focus, including mode of delivery, gestational age at delivery, preterm birth, premature rupture of membranes, placental abruption, postpartum hemorrhage, and intrauterine growth restriction (31, 33–37). Fetal and neonatal outcomes were also frequently reported, including birth weight, low birth weight, macrosomia, Apgar scores, neonatal anthropometric measures, respiratory distress syndrome, and neonatal intensive care unit admission (30, 33–38).

Recent trials have extended their focus beyond clinical endpoints to explore the biological and mechanistic pathways linking exercise to HDP risk. These pathways were mainly reflected in biomarkers of endothelial function, such as nitric oxide (NO) and endothelin-1 (ET-1) (27, 34), as well as metabolic markers, including total cholesterol, uric acid, insulin resistance, and lipid profile (30, 34, 35).

Moreover, some studies tended to incorporate outcomes related to maternal well-being, such as anxiety, depression, sleep quality, satisfaction, and maternal comfort during labor (33, 36, 37, 39).

In prevention-oriented studies involving women at high risk of developing HDP, three intervention studies directly examined HDP incidence (28, 30, 34). Khoram et al. reported that a walking intervention reduced the incidence of gestational hypertension and preeclampsia compared with control care (28). Similarly, Karthiga et al. found that yoga was associated with a substantially lower incidence of new-onset HDP (34). In contrast, Yeo et al. reported that stretching was associated with fewer cases of preeclampsia than walking, whereas gestational hypertension occurred more frequently in the stretching group; however, neither between-group difference reached statistical significance (30). Overall, these studies suggest that structured exercise may reduce HDP-related risk in high-risk pregnant women, although the effects may differ depending on the type of exercise performed. Blood pressure and cardiovascular regulation outcomes were also generally reported in a favorable direction (26, 28, 38). In the study by Khoram et al., the walking program was associated with improvements in both systolic and diastolic blood pressure (28). Similarly, Claiborne et al., using pooled data from three prospective randomized controlled trials, found that greater total prenatal exercise volume tended to be associated with lower systolic blood pressure across pregnancy among women at risk of HDP (38). Yeo et al. further reported lower resting heart rate and mean pulse pressure in the stretching group compared with the walking group (26). Yoga-based interventions also showed favorable effects on autonomic, endothelial, and inflammatory regulation (27, 34). Karthiga et al. reported increased heart rate variability and baroreflex sensitivity in the yoga group, alongside higher nitric oxide levels and lower interleukin-6 levels (34). Consistently, Zainiyah et al. found that prenatal yoga significantly reduced endothelin-1 and increased nitric oxide levels among pregnant women at high risk of preeclampsia, whereas the control group showed the opposite pattern (27). Additionally, Yeo et al. found that transferrin, an antioxidant-related marker, was significantly higher at labor in the stretching group than in the walking group (30). Regarding the pregnancy and neonatal outcomes, Karthiga et al. reported more favorable maternal and neonatal outcomes in the yoga group, including less painful delivery, shorter labor duration, higher neonatal birthweight, and improved Apgar scores (34). Claiborne et al. also reported that greater prenatal exercise volume was positively associated with gestational age at birth and birthweight among women at risk of HDP (38). However, Yeo et al. observed no significant between-group differences in birth outcomes between stretching and walking groups (30). Because the study lacked a non-exercise control group, it remains unclear whether either stretching or walking had an independent effect on birth outcomes compared with usual prenatal care (30).

In management-oriented studies involving women with established HDP, most studies reported favorable effects of exercise-based interventions on blood pressure control.

Awad et al. found that both stretching exercise and autogenic training reduced systolic and diastolic blood pressure among women with mild preeclampsia receiving methyldopa, although no significant between-group differences were observed (32). Makhija et al. also reported a significantly greater reduction in systolic blood pressure before delivery in the integrated yoga group among primigravid women with mild gestational hypertension in the third trimester (33). Chen et al. examined different exercise modalities (aerobic exercise, resistance training, and combined exercise training) in women with well-controlled HDP and improved morning blood pressure was observed in exercise groups, particularly the combined exercise training group (35). Yu et al. and Wang et al. similarly reported better blood pressure control following aerobic exercise combined with evidence-based care or systematic nursing care compared with routine or standard care (36, 37). Regarding HDP progression, Makhija et al. reported that, in primigravid women with mild gestational hypertension, there were fewer cases of progression to preeclampsia in the integrated yoga group than in the control group, but this difference did not reach statistical significance (33). Awad et al. reported reductions in proteinuria in both the stretching and autogenic training groups, but the absence of a usual-care-only control group limits the ability to determine whether these improvements were attributable to the non-pharmacological interventions themselves (32). Pregnancy, labor, and neonatal outcomes were reported inconsistently across management-oriented studies. Makhija et al. found that integrated yoga was associated with a shorter first stage and total duration of labor, as well as higher maternal comfort during early labor, but no significant between-group differences were observed in neonatal outcomes, including birthweight, preterm birth, fetal growth restriction, or Apgar scores (33). In contrast, Chen et al. also reported favorable pregnancy outcomes, particularly in the combined aerobic and resistance training group, including a lower preterm birth rate, a lower incidence of premature rupture of membranes, and higher Apgar scores (35). Yu et al. reported fewer adverse pregnancy outcomes, including preterm delivery, cesarean delivery, placental abruption, postpartum hemorrhage, fetal distress, and neonatal asphyxia, following evidence-based care combined with moderate aerobic exercise (36). Wang et al. similarly reported a higher vaginal delivery rate and improved neonatal outcomes, including higher birthweight and Apgar scores, following aerobic exercise combined with systematic nursing care (37). Psychological well-being and sleep outcomes have also been improved. Yu et al. reported lower anxiety and depression scores and longer sleep duration in the evidence-based care plus aerobic exercise group compared with routine care alone (36). Wang et al. also reported greater reductions in anxiety and depression scores in the aerobic exercise plus systematic nursing care group compared with standard care (37).

Importantly, although the above studies suggest that exercise interventions may have potential for improving maternal and neonatal health and for preventing or adjunctively managing HDP, their findings should be interpreted with caution because several studies used statistical approaches that may not have fully accounted for their pre–post or repeated-measures designs (28, 32, 33, 35–37).

## 4 Discussion

This scoping review identified a small but clinically diverse body of literature on structured exercise interventions for HDP. The current evidence base remains highly heterogeneous in terms of participant characteristics, intervention design, intensity regulation, safety procedures, and outcome selection. The overall findings suggest that structured exercise interventions may have favorable potential for improving maternal and neonatal health. However, these findings should be interpreted with caution because of the heterogeneity and methodological limitations of the available studies.

The studies included in this review primarily involved two distinct clinical contexts: (1) prevention-oriented interventions in women at elevated risk of HDP, and (2) management-oriented interventions in women with diagnosed HDP. These populations differ substantially in baseline cardiovascular status, obstetric risk, monitoring requirements, and exercise tolerance, which likely influences both intervention selection and treatment response (12). Exercise should not be conceptualized as a uniform intervention across the HDP spectrum. The risk-benefit profile of exercise interventions in women at elevated risk of HDP differs substantially from that in women with established HDP (19, 24, 25, 33). Therefore, future research should develop more stratified exercise recommendations according to disease stage, severity, and risk profile.

Aerobic exercise was more commonly used in included studies and has been associated with improved blood pressure control, vascular perfusion, endothelial function, and maternal hemodynamic regulation (40, 41). Lower-load interventions such as stretching and yoga were also used frequently (27, 32–34). These approaches may be favored because they impose lower cardiovascular demands while still offering potential benefits through reductions in sympathetic activation, peripheral vascular resistance, and psychological stress (32, 33). Notably, although resistance exercise is generally considered acceptable in healthy pregnant women (24), it has been used more cautiously in HDP-related populations. In this review, only two studies incorporated resistance exercise either alone or in combination with aerobic exercise, and limited participation to women with well-controlled blood pressure (35, 38). Future studies using resistance exercise interventions in women at risk of HDP or with established HDP should clearly define eligibility criteria for participation, specify the intensity and loading parameters of the resistance exercise protocol, report intervention-induced blood pressure responses and acceptable blood pressure ranges, and establish explicit symptom-triggered cessation criteria and supervision procedures.

A major finding of this review is that current evidence remains insufficient to establish a clear FITT-VP-based framework (frequency, intensity, time, type, volume, and progression) for women with HDP (42). Substantial variation existed across studies in training frequency, session duration, intervention length, comparator conditions, and progression strategies. Importantly, exercise intensity was particularly inconsistently defined and classified across the included studies. For example, one study prescribed exercise at a heart rate 20% above resting values, with an upper limit of 140 beats/min, but described the intervention as low intensity (31). Another study described the intervention as moderate-intensity exercise but used a fixed target heart-rate range of 100–120 beats/min (37). A further study described the intervention as moderate intensity but reported a very broad heart-rate range of 100–200 beats/min (36), limiting interpretability. In addition, several yoga or stretching studies did not report explicit intensity classifications or objective intensity indicators; therefore, their intensity was inferred as low intensity based on the intervention type and content (27, 32–34). These inconsistencies highlight an important methodological limitation in this field: exercise intensity is often insufficiently standardized and poorly operationalized. Future studies should clearly define exercise intensity using standardized and reproducible criteria, report both the intended intensity category and the specific monitoring methods used.

A recent consensus statement from the American College of Sports Medicine (ACSM) and Exercise and Sport Science Australia (ESSA) emphasized that traditional fixed markers, such as percentage of maximal heart rate or heart rate reserve, often fail to produce comparable physiological stress across individuals, with greater emphasis now placed on the use of RPE as an important adjunct tool for monitoring exercise intensity (43). In pregnant women, heart rate responses may be influenced by gestational age, plasma volume expansion, autonomic nervous system, stress, and medication use (44–46). Therefore, future exercise protocols for pregnant populations should not rely on fixed heart rate limits alone. Instead, intensity monitoring should use a multiple-metric approach combining RPE and the talk test, supplemented by symptom monitoring and, where feasible, individualized baseline assessment (45, 47).

Explicit exercise intervention termination criteria or warning symptoms requiring cessation were reported in only a minority of studies (26, 28, 30, 36, 39). Beyond general precautions for exercise during pregnancy, such as maintaining adequate hydration and avoiding excessive heat stress, studies involving women with hypertensive disorders of pregnancy should provide clearer reporting of monitoring procedures (25). Inadequate reporting of these procedures may reduce reproducibility, hinder clinical translation, and limit confidence among clinicians and pregnant women. For HDP populations, safety monitoring should be treated as a core intervention component. Future HDP trials should clearly report eligibility-related blood pressure thresholds, pre- and post-intervention blood pressure assessment, symptom monitoring during exercise, adverse-event definitions, cessation criteria, and referral or discontinuation pathways. Where feasible, home blood pressure monitoring should also be incorporated to capture blood pressure trajectories beyond supervised intervention sessions. Fetal monitoring should also be reported when clinically indicated. In addition, future studies should report supervision, adherence, intervention fidelity, medication use, and obstetric co-interventions, as these factors directly influence intervention feasibility, reproducibility, and clinical interpretation.

Overall, the research findings suggest that exercise may promote maternal and neonatal health and improve pregnancy outcomes. However, these findings should be interpreted with caution, as several studies primarily relied on conventional and separate between-group and pre-post comparisons, such as t tests and *χ*^2^ tests, with limited use of model-based analyses capable of accounting for baseline differences, repeated measurements, group-by-time interactions, and multiple comparisons (28, 32, 33, 35–37). Additionally, most interventions in this field have been evaluated only through pregnancy or up to delivery, with limited attention to postpartum cardiovascular recovery and longer-term maternal outcomes (48). This is an important gap because HDP is increasingly recognized as a female-specific cardiovascular risk marker, and pregnancy may serve as a cardiovascular stress test that identifies women at increased risk of later-life hypertension and cardiovascular disease (49–51). Future studies should therefore incorporate postpartum cardiovascular outcomes, including persistent hypertension, home or ambulatory blood pressure trajectories, arterial stiffness, endothelial function, cardiometabolic risk markers, and transition from obstetric to cardiovascular follow-up care.

This review has several limitations. First, as a scoping review, it was intended to map the extent, characteristics, and research gaps of the available literature rather than to quantitatively determine the effectiveness of specific interventions; therefore, no pooled conclusions regarding intervention efficacy can be drawn. Second, substantial heterogeneity in HDP definitions, participant risk profiles, intervention prescriptions, comparator conditions, and outcome measures limited cross-study comparability. Third, co-interventions such as antihypertensive medications, nutritional counselling, and nursing care were reported inconsistently, making it difficult to isolate the independent contribution of exercise interventions. Fourth, this review included only peer-reviewed studies published in English. Although clinical trial registries were searched as supplementary sources, registered but unpublished trials, ongoing studies, and studies published in languages other than English were not included in the analysis. Therefore, this review may have publication and language bias. Overall, the findings of this review should primarily be interpreted as an overview of the current evidence landscape and research gaps, rather than as definitive guidance for physical activity in women with HDP.

## 5 Conclusions

Current evidence on structured exercise interventions related to HDP remains limited. Existing studies are characterized by a small number of trials, inconsistency in exercise intervention protocols, non-standardized and poorly operationalized definitions of exercise intensity, insufficient safety monitoring and reporting, and a lack of long-term follow-up. Available evidence remains insufficient to establish HDP-specific exercise prescriptions tailored to different clinical profiles. Future research should more clearly define target populations, develop standardized exercise intervention prescriptions tailored to participants’ clinical characteristics and risk profiles, implement more rigorous intervention delivery, safety monitoring, adherence assessment procedures, and apply appropriate statistical methods. These efforts will provide a stronger foundation for direct comparisons across different exercise modalities and doses, thereby facilitating the development of scientifically grounded, standardized, and clinically applicable recommendations.

## Data Availability

No new primary data were generated in this scoping review. The data analyzed were obtained from the previously published studies included in the review, and the extracted and synthesized information supporting the conclusions is presented in the article. Further inquiries can be directed to the corresponding author.
