# Supplementary material for: Structured exercise interventions for women at high risk of or diagnosed with hypertensive disorders of pregnancy: a scoping review
Source: Front Cardiovasc Med. 2026 Jul 21;13:1876680. doi: 10.3389/fcvm.2026.1876680 (PMC13433281; doi:10.3389/fcvm.2026.1876680)
Supplement: Supplementary file 1 [file Supplementaryfile1.docx]

***Supplementary Material***

**Supplementary material 1. Preferred Reporting Items for Systematic Reviews and Meta-Analyses extension for Scoping Reviews (PRISMA-ScR) checklist**

| **SECTION** | **ITEM** | **PRISMA-ScR CHECKLIST ITEM** | **Location** |
| --- | --- | --- | --- |
| **TITLE** | | | |
| Title | 1 | Identify the report as a scoping review. | Title |
| **ABSTRACT** | | | |
| Structured summary | 2 | Provide a structured summary that includes (as applicable): background, objectives, eligibility criteria, sources of evidence, charting methods, results, and conclusions that relate to the review questions and objectives. | Abstract |
| **INTRODUCTION** | | | |
| Rationale | 3 | Describe the rationale for the review in the context of what is already known. Explain why the review questions/objectives lend themselves to a scoping review approach. | Introduction |
| Objectives | 4 | Provide an explicit statement of the questions and objectives being addressed with reference to their key elements (e.g., population or participants, concepts, and context) or other relevant key elements used to conceptualize the review questions and/or objectives. | Introduction |
| **METHODS** | | | |
| Protocol and registration | 5 | Indicate whether a review protocol exists; state if and where it can be accessed (e.g., a Web address); and if available, provide registration information, including the registration number. | Methods |
| Eligibility criteria | 6 | Specify characteristics of the sources of evidence used as eligibility criteria (e.g., years considered, language, and publication status), and provide a rationale. | Eligibility criteria |
| Information sources* | 7 | Describe all information sources in the search (e.g., databases with dates of coverage and contact with authors to identify additional sources), as well as the date the most recent search was executed. | Methods |
| Search | 8 | Present the full electronic search strategy for at least 1 database, including any limits used, such that it could be repeated. | Supplementary Material S2 |
| Selection of sources of evidence† | 9 | State the process for selecting sources of evidence (i.e., screening and eligibility) included in the scoping review. | Eligibility criteria |
| Data charting process‡ | 10 | Describe the methods of charting data from the included sources of evidence (e.g., calibrated forms or forms that have been tested by the team before their use, and whether data charting was done independently or in duplicate) and any processes for obtaining and confirming data from investigators. | Eligibility criteria |
| Data items | 11 | List and define all variables for which data were sought and any assumptions and simplifications made. | Eligibility criteria |
| Critical appraisal of individual sources of evidence§ | 12 | If done, provide a rationale for conducting a critical appraisal of included sources of evidence; describe the methods used and how this information was used in any data synthesis (if appropriate). | Not conducted |
| Synthesis of results | 13 | Describe the methods of handling and summarizing the data that were charted. | Eligibility criteria |
| **RESULTS** | | | |
| Selection of sources of evidence | 14 | Give numbers of sources of evidence screened, assessed for eligibility, and included in the review, with reasons for exclusions at each stage, ideally using a flow diagram. | Figure 1 |
| Characteristics of sources of evidence | 15 | For each source of evidence, present characteristics for which data were charted and provide the citations. | Characteristics of included articles |
| Critical appraisal within sources of evidence | 16 | If done, present data on critical appraisal of included sources of evidence (see item 12). | Not conducted |
| Results of individual sources of evidence | 17 | For each included source of evidence, present the relevant data that were charted that relate to the review questions and objectives. | Table 1, 2, 3 |
| Synthesis of results | 18 | Summarize and/or present the charting results as they relate to the review questions and objectives. | Characteristics of included participants; Characteristics of structured exercise  Interventions; Characteristics of outcomes and  findings |
| **DISCUSSION** | | | |
| Summary of evidence | 19 | Summarize the main results (including an overview of concepts, themes, and types of evidence available), link to the review questions and objectives, and consider the relevance to key groups. | Discussion |
| Limitations | 20 | Discuss the limitations of the scoping review process. | Discussion |
| Conclusions | 21 | Provide a general interpretation of the results with respect to the review questions and objectives, as well as potential implications and/or next steps. | Conclusions |
| **FUNDING** | | | |
| Funding | 22 | Describe sources of funding for the included sources of evidence, as well as sources of funding for the scoping review. Describe the role of the funders of the scoping review. | Funding |

**Supplementary material 2. Search strategy**

**Search strategy in Web of Science**

1. Hypertension, Pregnancy Induced (Topic) or Hypertension, Pregnancy Induced (Topic) or Pregnancy-Induced Hypertension (Topic) or Gestational Hypertension (Topic) or Hypertension, Gestational (Topic) or Pregnancy Induced Hypertension (Topic) or Hypertensions, Pregnancy Induced (Topic) or Hypertensions, Pregnancy Induced (Topic) or Induced Hypertensions, Pregnancy (Topic) or Transient Hypertension, Pregnancy (Topic) or Hypertension, Pregnancy Transient (Topic) or Pregnancy Transient Hypertension (Topic) or Pre-Eclampsia (Topic) or Pre Eclampsia (Topic) or Preeclampsia (Topic) or Pregnancy Toxemias (Topic) or Pregnancy Toxemia (Topic) or Toxemia, Pregnancy (Topic) or Edema-Proteinuria-Hypertension Gestosis (Topic) or Edema Proteinuria Hypertension Gestosis (Topic) or Gestosis, Edema-Proteinuria-Hypertension (Topic) or Hypertension-Edema-Proteinuria Gestosis (Topic) or Gestosis, Hypertension-Edema-Proteinuria (Topic) or Hypertension Edema Proteinuria Gestosis (Topic) or Proteinuria-Edema-Hypertension Gestosis (Topic) or Gestosis, Proteinuria-Edema-Hypertension (Topic) or Proteinuria Edema Hypertension Gestosis (Topic) or Toxemias, Pregnancy (Topic) or Toxemia Of Pregnancy (Topic) or Eclampsia (Topic) or Eclampsias (Topic)
2. Exercise (Topic) or Physical Exercise (Topic) or Aerobic Exercise (Topic) or Isometric Exercise (Topic) or Pregnancy Toxemia (Topic) or Exercise Training (Topic) or Physical Activity (Topic) or Walking (Topic) or Resistance Training (Topic) or Yoga (Topic)
3. #1 AND #2

**Search strategy in PubMed**

1. (((((((((((((("Hypertension, Pregnancy-Induced"[Mesh]) OR (Hypertension, Pregnancy Induced[Title/Abstract])) OR (Pregnancy-Induced Hypertension[Title/Abstract])) OR (Gestational Hypertension[Title/Abstract])) OR (Hypertension, Gestational[Title/Abstract])) OR (Pregnancy Induced Hypertension[Title/Abstract])) OR (Hypertensions, Pregnancy Induced[Title/Abstract])) OR (Induced Hypertension, Pregnancy[Title/Abstract])) OR (Induced Hypertensions, Pregnancy[Title/Abstract])) OR (Transient Hypertension, Pregnancy[Title/Abstract])) OR (Hypertension, Pregnancy Transient[Title/Abstract])) OR (Pregnancy Transient Hypertension[Title/Abstract])) OR ((((((((((((((((("Pre-Eclampsia"[Mesh]) OR (Pre Eclampsia[Title/Abstract])) OR (Pre-Eclampsia[Title/Abstract])) OR (Pregnancy Toxemias[Title/Abstract])) OR (Pregnancy Toxemia[Title/Abstract])) OR (Toxemia, Pregnancy[Title/Abstract])) OR (Edema-Proteinuria-Hypertension Gestosis[Title/Abstract])) OR (Edema Proteinuria Hypertension Gestosis[Title/Abstract])) OR (Gestosis, Edema-Proteinuria-Hypertension[Title/Abstract])) OR (Hypertension-Edema-Proteinuria Gestosis[Title/Abstract])) OR (Gestosis, Hypertension-Edema-Proteinuria[Title/Abstract])) OR (Hypertension Edema Proteinuria Gestosis[Title/Abstract])) OR (Proteinuria-Edema-Hypertension Gestosis[Title/Abstract])) OR (Gestosis, Proteinuria-Edema-Hypertension[Title/Abstract])) OR (Proteinuria Edema Hypertension Gestosis[Title/Abstract])) OR (Toxemias, Pregnancy[Title/Abstract])) OR (Toxemia Of Pregnancy[Title/Abstract]))) OR ((("Eclampsia"[Mesh]) OR (Eclampsia[Title/Abstract])) OR (Eclampsias[Title/Abstract]))) AND (((((((((((("Exercise"[Mesh]) OR (Exercise[Title/Abstract])) OR (Physical Exercise[Title/Abstract])) OR (Aerobic Exercise[Title/Abstract])) OR (Isometric Exercise[Title/Abstract])) OR (Exercise Training[Title/Abstract])) OR (Physical Activity[Title/Abstract])) OR (Walking[Title/Abstract])) OR (Running[Title/Abstract])) OR (Resistance Training[Title/Abstract])) OR (Yoga[Title/Abstract])) OR ((("Exercise Therapy"[Mesh]) OR (Exercise Therapy[Title/Abstract])) OR (Exercise Intervention[Title/Abstract])))

**Search strategy in Cochrane Library**

1. [Hypertension, Pregnancy-Induced] explode all trees
2. (Hypertension, Pregnancy Induced or Pregnancy-Induced Hypertension or Gestational Hypertension or Hypertension, Gestational or Pregnancy Induced Hypertension or Hypertensions, Pregnancy Induced or Induced Hypertension, Pregnancy or Induced Hypertensions, Pregnancy or Transient Hypertension, Pregnancy or Hypertension, Pregnancy Transient or Pregnancy Transient Hypertension):ti,ab,kw.
3. [Pre-Eclampsia] explode all trees
4. [Eclampsia] explode all trees
5. (Pre Eclampsia or Preeclampsia or Pregnancy Toxemias or Pregnancy Toxemia or Toxemia, Pregnancy or Edema-Proteinuria-Hypertension Gestosis or Edema Proteinuria Hypertension Gestosis or Gestosis, Edema-Proteinuria-Hypertension or Hypertension-Edema-Proteinuria Gestosis or Gestosis, Hypertension-Edema-Proteinuria or Hypertension Edema Proteinuria Gestosis or Proteinuria-Edema-Hypertension Gestosis or Gestosis, Proteinuria-Edema-Hypertension or Proteinuria Edema Hypertension Gestosis or Toxemias, Pregnancy or Toxemia Of Pregnancy):ti,ab,kw
6. (Eclampsias):ti,ab,kw
7. #1 or #2 or #3 or #4 or #5 or #6
8. [Exercise] explode all trees
9. (Exercise or Physical Exercise or Aerobic Exercise or Isometric Exercise or Exercise Training or Physical Activity or Walking or Resistance Training or Yoga):ti,ab,kw
10. #8 or #9
11. #7 and #10

**Search strategy in Embase**

1. 'maternal hypertension'/exp
2. 'maternal hypertension':ab,ti OR 'gestational hypertension':ab,ti OR 'hypertension in pregnancy':ab,ti OR 'hypertension induced by pregnancy':ab,ti OR 'hypertension, maternal':ab,ti OR 'hypertension, pregnancy induced':ab,ti OR 'hypertension, pregnancy-induced':ab,ti OR 'hypertensive disorder of pregnancy':ab,ti OR (pih:ab,ti AND 'pregnancy induced hypertension':ab,ti) OR 'pregnancy associated hypertension':ab,ti OR 'pregnancy hypertension':ab,ti OR 'pregnancy induced hypertension':ab,ti OR 'pregnancy-induced hypertension':ab,ti
3. 'preeclampsia'/exp
4. 'eclamptic toxaemia':ab,ti OR 'eclamptic toxemia':ab,ti OR 'eclamptogenic toxaemia':ab,ti OR 'eclamptogenic toxemia':ab,ti OR 'edema-proteinuria-hypertension gestoses':ab,ti OR 'edema-proteinuria-hypertension gestosis':ab,ti OR 'eph gestoses':ab,ti OR 'eph gestosis':ab,ti OR 'eph syndrome':ab,ti OR 'eph toxemia':ab,ti OR 'gestational toxaemia':ab,ti OR 'gestational toxemia':ab,ti OR 'gestational toxicosis':ab,ti OR gestoses:ab,ti OR gestosis:ab,ti OR 'gestosis, eph':ab,ti OR 'hep syndrome':ab,ti OR 'maternal toxemia':ab,ti OR 'pre eclampsia':ab,ti OR 'pre eclamptic':ab,ti OR 'pre-eclamptic toxaemia':ab,ti OR 'pre-eclamptic toxemia':ab,ti OR preclampsia:ab,ti OR preeclamptic:ab,ti OR 'preeclamptic toxaemia':ab,ti OR 'preeclamptic toxemia':ab,ti OR 'pregnancy toxaemia':ab,ti OR 'pregnancy toxaemias':ab,ti OR 'pregnancy toxemia':ab,ti OR 'pregnancy toxemias':ab,ti OR 'pregnancy toxicosis':ab,ti OR 'proteinuric hypertension of pregnancy':ab,ti OR 'toxaemia gravidum':ab,ti OR 'toxaemia, preeclamptic':ab,ti OR 'toxemia during pregnancy':ab,ti OR 'toxemia gravidum':ab,ti OR 'toxemia in pregnancy':ab,ti OR 'toxemic pregnancy':ab,ti OR 'toxicosis gravidarum':ab,ti OR preeclampsia:ab,ti
5. 'eclampsia'/exp
6. eclamptic:ab,ti OR 'eclamptic convulsion':ab,ti OR 'eclamptic convulsions':ab,ti OR 'eclamptic fits':ab,ti OR 'eclamptic seizure':ab,ti OR 'eclamptic seizures':ab,ti OR eclampsia:ab,ti
7. 'exercise'/exp
8. exercise:ab,ti OR 'exercise training':ab,ti OR 'fitness training':ab,ti OR 'fitness workout':ab,ti OR 'physical conditioning, human':ab,ti OR 'physical exercise':ab,ti OR 'physical work-out':ab,ti OR 'physical workout':ab,ti
9. 'physical activity'/exp
10. 'physical activity':ab,ti OR walking:ab,ti OR yoga:ab,ti OR 'resistence training':ab,ti

11	#1 OR #2 OR #3 OR #4 OR #5 OR #6

12	#7 OR #8 OR #9 OR #10

13	#11 AND #12

**Search strategy in ClinicalTrials.gov**

(preeclampsia OR pre-eclampsia OR gestational hypertension OR pregnancy-induced hypertension OR hypertensive disorders of pregnancy OR eclampsia) and (exercise OR physical activity OR training OR physical exercise OR walking OR cycling OR yoga OR stretching OR resistance training)

**Search strategy in World Health Organization International Clinical Trials Registry Platform (WHO ICTRP)**

(preeclampsia OR pre-eclampsia OR gestational hypertension OR pregnancy-induced hypertension OR hypertensive disorders of pregnancy OR eclampsia) and (exercise OR physical activity OR training OR physical exercise OR walking OR cycling OR yoga OR stretching OR resistance training)
